# Effect of CYP3 A4, CYP3 A5 and ABCB1 gene polymorphisms on the clinical efficacy of tacrolimus in the treatment of nephrotic syndrome

**DOI:** 10.1186/s40360-018-0202-9

**Published:** 2018-04-03

**Authors:** Min Li, Min Xu, Wei Liu, Xin Gao

**Affiliations:** 1 Department of Nephrology, The 88th Hospital of PLA, Taian, People’s Republic of China; 2 Department of Medicine, The 88th Hospital of PLA, Taian, People’s Republic of China

**Keywords:** CYP3 A4*1G, CYP3 A5*3, ABCB1C1236T, ABCB1 G2677 T/A, TAC, Clinical efficacy

## Abstract

**Background:**

The efficacy of tacrolimus (TAC) is variable in the treatment of nephrotic syndrome (NS), which might be related to genetic variation among patients. Therefore, we aim to investigate the effects of CYP3 A4, CYP3 A5 and ABCB1 gene polymorphisms on the clinical efficacy of TAC in the treatment of NS patients.

Methods 100 NS patients were treated with TAC and prednisone and followed up for 3 months. Genotype differences (CYP3 A4*1G, CYP3 A5*3, ABCB1 1236C > T and ABCB1 2677G > T/A) were detected by Sanger sequencing. The clinical efficacy was evaluated by the 24 h urinary protein quantitation, albumin, renal function and the degree of edema. Multivariable logistic regression was used to analyze the effect of gene polymorphisms on the clinical efficacy of TAC.

**Results:**

There were 35 patients (35%) with complete remission, 43 patients (43%) with partial remission, 22 patients (22%) without remission, and no patients with recurrence. For CYP3A4, there were 56, 42, and 2 patients with *1/*1, *1/*1G and *1G/*1G genotype, respectively. For CYP3A5, there were 8, 36 and 56 cases with*1/*1, *1/*3 and *3/*3 genotype, respectively. For ABCB1 C1236T, there were 10, 44, and 46 cases with 1236CC, 1236CT and 1236TT genotype, respectively. For ABCB1 G2677 T/A, there were 13, 57, and 30 patients with 2677GG genotype, 2677GT/GA genotype and 2677TT/AA/TA genotype, respectively. The mutant allele frequencies of CYP3A4*1G, CYP3A5*3, ABCB1 C1236T and ABCB1 G2677 T/A were 23%, 74%, 68% and 58.5%, respectively. Results reveal that the gene polymorphisms of CYP3A4 and CYP3A5 and CCB do not affect the clinical efficacy of TAC. For ABCB1 C1236T,TT genotype can increase the effectiveness 12.085 times compared with CC and CT genotype (*P* = 0.018, OR = 12.085, 95%CI 1.535–95.148). For ABCB1 G2677 T/A, the clinical efficacy of patients with mutant genotype is 8.683 times than that of wild-type and heterozygous patients (*P* = 0.042, OR = 8.683, 95%CI 1.080–69.819). Overweight patients can improve the clinical efficacy by 15.838 times (*P* = 0.020, OR = 15.838, 95%CI1.550–161.788).

**Conclusions:**

ABCB1 C1236T, ABCB1 G2677 T/A genotype and BMI are probably the factors influencing the clinical efficacy of TAC in treating patients with NS.

**Electronic supplementary material:**

The online version of this article (10.1186/s40360-018-0202-9) contains supplementary material, which is available to authorized users.

## Background

Nephrotic syndrome (NS) is a common condition in renal medicine which has high morbidity in middle aged and elderly people. The response to treatment varies greatly in NS patients, and some of them may develop into renal failure. Tacrolimus (TAC), an inhibitor of calcium phosphatase, also named FK506, is used in the treatment of NS in recent years [1, 2]. The pharmacokinetics of TAC is variable among individuals. The safety range between effective dose and toxic dose is narrow, and it is easy to be affected by gene polymorphisms and other combined drugs. Therefore, it is very important to monitor the blood concentration of TAC in clinical treatment. Drug genomics studies showed that TAC was mainly metabolized by drug metabolism enzyme CYP3A4/5, and then transported by the P glycoprotein (P-gp) [3, 4]. Therefore, CYP3A4, CYP3A5 and P-gp gene polymorphisms are responsible for individual differences in clinical efficacy of TAC [5, 6]. Specifically, the National Health and Family Planning Commission of People’s Republic of China gives a formula for TAC dose in Chinese population [4].

The CYP3 A4*1G (rs2242480) is the major site of the single nucleotide polymorphisms (SNPs) and closely related to the metabolism of TAC. The mutation occurs at the 10th intron (G > A; the wild type: CYP3 A4*1/*1; the heterozygous type: CYP3 A4*1/*1G; the mutant type: CYP3 A4*1G/*1G) [5, 7, 8]. The CYP3 A5 also has multipled SNPs and most attention has been paid to the site of CYP3 A5*3 (rs776746). The mutation occurs at the 3rd intron (A > G; the wild type: CYP3 A5 *1/*1; the heterozygous type: CYP3 A5*1/*3; the mutant type: CYP3 A5*3/*3) [9, 10]. ABCB1 gene, which encodes P-gp, is also known as multidrug resistance gene. The two most common SNPs in the protein coding region are rs1128503 (1236C > T; the wild type: ABCB1 1236 CC; the heterozygous type: ABCB1 1236 CT; the mutant type: ABCB1 1236 TT)) and rs2032582 (2677G > T/A; the wild type: ABCB1 2677GG; the heterozygous type: ABCB1 2677GT/GA; the mutant type: ABCB1 2677TT/AA/TA) [11, 12]. The existing literature investigating these gene polymorphisms have been mainly derived from cohorts of renal transplant patients. There is a widespread view that CYP3A5 nonexpressers (CYP3 A5*3/*3 carriers) require lower mean TAC doses and exhibit higher concentration/dose ratios in renal transplant patients [3, 13]. However, the effect of the CYP3A4*1G, ABCB1 1236C > T and 2677G > T/A genetic polymorphisms on TAC pharmacokinetics in renal transplant recipients is controversial [12, 14]. As for NS patient, little is known about these gene polymorphisms in the clinical efficacy of TAC.

Therefore, in order to guide the rational use of clinical medicine, we further investigate the effect of CYP3 A4, CYP3 A5 and ABCB1 gene polymorphisms on the clinical efficacy of patients with NS in Chinese population in this study.

## Methods

### Object of study

A total of 100 NS patients with a mean age of 37.28 ± 17.0 years between January and November 2016 were included in this study. They all live in or around Tai’an. NS aetiology was determined by renal biopsy. Glomerular filtration rate (GFR) was greater than 80 mL/min/1.73m^2^ in all patients. 18.5–25 kg/m^2^ is considered to be the normal range for BMI in adults, and 6–19 kg/m^2^ in children. Urine routine, 24 h urinary protein, blood routine, liver function, renal function, blood lipid and blood sugar were also detected.

### Ethical consideration

We received approval from the Medical Ethics Committee of the 88th Hospital of PLA (Chinese People’s Liberation Army) for undertaking this study. The study was designed to be secure and fair to patients while minimizing risk of harm to participants. The included participants provided written informed voluntary consent and participants under the age of 18 years had written consent obtained from their parents. Participants had the right to withdraw from the study at any time.

### Therapeutic regimen

All the patients were treated with TAC and prednisone. TAC was given at the dose of 0.05 mg kg^− 1^ day^− 1^ for both adults and children, divided twice from oral application at intervals of 12 h [3, 15–17]. Prednisone was used 0.5 mg/kg daily initially. After 8 weeks of treatment, the amount of prednisone was reduced by 5 mg every two weeks to 20 mg. Then, the dose of 20 mg/d was maintained for 2 months and the rest of the prednisone gradually reduced to complete withdrawal [3].

### Evaluation of safety

Evaluation of safety is to observe the safety of the TAC in the study. Referring to the relevant literatures, the main side effects of TAC were closely observed in the present study, including abnormality of glucose tolerance, infection, renal toxicity, gastrointestinal adverse reactions, elevated blood pressure and liver toxicity, etc. Once the uncontrollable adverse reactions are found, we should stop TAC immediately.

### Clinical evaluation criteria

The patients were followed up for 3 months. The clinical efficacy was evaluated by the 24 h urinary protein quantitation, albumin (ALB), renal function and the degree of edema. Evaluation criteria are divided into complete remission (CR), partial remission (PR), no remission (NR) and recrudescence [6, 9]. (1) CR: Urine protein < 0.3 g/d, ALB > 35 g/L, edema disappeared and stable renal function. (2) PR: Urine protein 0.3 ~ 3.5 g/d or decreased > 50%, ALB > 30 g/L, edema disappeared and stable renal function. (3) NR: Urine protein > 3.5 g/d and ALB < 30 g/L with edema or deterioration of renal function. (4) Recrudescence: When reaching CR or PR, proteinuria > 3.5 g/d and ALB < 30 g/L, accompanied by edema or deterioration of renal function appear again. CR + PR are considered to be effective and NR + recurrence are treated as ineffective (Table 1).

**Table 1:** Assignment of logistic regression analysis

Factors	Assignment description
CYP3 A4	CYP3 A4*1/*1 = 0, CYP3 A4*1/*1G + CYP3 A4*1G/*1G = 1
CYP3 A5	CYP3 A5*1/*1+ CYP3 A5*1/*3 = 0,CYP3 A5*3/*3 = 1
ABCB1 C1236T	CC + CT = 0,TT = 1
ABCB1 G2677 T/A	GG + GT + GA = 0,TT + TA + AA = 1
CCB	Not applied CCB = 0, applied CCB = 1
BMI	the normal range = 0, the abnormal range that is overweight =1

### Gene determination and sequencing

Blood was collected in heparin anticoagulation tube, and then DNA was extracted (Invitrogen, Carlsbad, CA, USA). Sanger sequencing was used to detect the genotypes of CYP3 A4*1G, CYP3 A5*3, ABCB1 C1236T and ABCB1 G2677 T/A. PCR amplification conditions were as follows: pre-denaturation at 94 °C for 2 min, denaturation at 94 °C for 30 s, anneal at 60 °C for 30 s, and extension at 72 °C for 20 s for a total of 38 cycles. At last, extension was performed at 72 °C for 2 min [7]. The primer F, primer R and the purified DNA were delivered to Shenzhen Hua Da Technology Company for sequencing.

### Sequencing map analysis

After all the samples were sequenced, the National Center for Biotechnology Information was used to search the sequencing map of CYP3A4*1G (Additional file 1: Fig. S1), CYP3A5*3 (Additional file 2: Fig. S2), ABCB1 C1236T (Additional file 3: Fig. S3) and G2677 T/A (Additional file 4: Fig. S4). CYP3 A4 genes were sequenced by forward sequencing method. The genes of CYP3 A5, ABCB1 C1236T and ABCB1 G2677 T/A were sequenced by reverse sequencing method.

### Statistical analysis

SPSS 18 software was used for statistical analysis. Concordance of genotype distribution with Hardy-Weinberg equilibrium was assessed using the χ^2^ test. Logistic regression analysis was used to compare the effects of CYP3 A4, CYP3 A5, ABCB1 C1236T, ABCB1 G2677 T/A, CCB and BMI on the clinical efficacy of TAC in the treatment of NS patients. We used the enter method, that was, the full model method, and all variables were entered into the equation to screen meaningful variables. Clinical efficacy was used as dependent variable and the following indicators were used as covariates (Table 1).

## Results

### Characteristics of patients

The characteristics of patients were given in Table 2. There were 10 cases of children. Among them, one children was 6 years old and the rest were between 15 and 18 years old. The average age were 15.90 ± 3.54 years, and the average weight were 49.4 ± 11.7 kg. NS could be divided into a variety of different pathological types. The population of this study included membranous nephropathy (MN) (36 cases, 36%), mesangial proliferative glomerulonephritis (MsPGN) (11 cases, 11%), minimal change nephropathy (MCN) (15 cases, 15%), focal segmental glomerulosclerosis (FSGS) (5 cases, 5%), systemic lupus erythematosus (SLE) (16 cases, 16%) and Henoch - Schonlein purpura nephritis (HSPN) (7 cases, 7%).

**Table 2:** Characteristics of patients

Variable	Value
Age, range (mean ± SD)	6–63 (37.28 ± 17.0)
Children/adults	10/ 90
Females, n (%)	25 (53.2)
Weight (kg), range (mean ± SD)	33.5–103 (63.5 ± 11.3)
BMI (kg/m^2^), range (mean ± SD)	19.0–33.6 (23.9 ± 2.8)
GFR (mL/min/1.73m^2^), range (mean ± SD)	83.4–177.0 (115 ± 34.9)
Using CCB (cases)(%)	12 (12%)

BMI: body mass index; GFR: glomerular filtration rate

### Clinical efficacy of TAC

There were 35 patients (35%) with CR, 43 patients (43%) with PR, 22 patients (22%) with NR, and no patients with recurrence. The overall rate of effective treatment was 78% (78/100) (Table 3).

**Table 3:** Remission rate of different diseases

Disease types	CR (cases)	PR (cases)	NR (cases)	Total	Effective rate (%)
MN	9	17	10	36	72.2
MsPGN	6	4	1	11	90.9
MCN	10	4	1	15	93.3
FSGS	0	0	5	5	0
SLE	5	16	5	26	80.7
HSPN	5	2	0	7	100
Total	35	43	22	100	78% (78/100)

MN: membranous nephropathy; MsPGN: mesangial proliferative glomerulonephritis; MCN: minimal change nephropathy; FSGS: focal segmental glomerulosclerosis; SLE: systemic lupus erythematosus; HSPN: Henoch - Schonlein purpura nephritis;

The outcomes of patient with different pathological causes were as follows. For MN, there were 9 patients with CR, 17 patients with PR, and 10 cases with NR. In MsPGN, the number of patients with CR, PR, and NR was 6, 4 and 1, respectively. There were 15 people in the MCN group, including 10 cases of CR, 4 cases of PR, and 1 case with NR. All of 5 patients with FSGS were not relieved. As for SLE, the number of people was 5,16,5 in CR, PR, and NR groups, respectively. There were 5 patients with CR and 2 cases with PR among 7 patients with HSPN.

### Genetic equilibrium test

For CYP3A4, there were 56 patients with *1/*1 genotype, 42 patients with *1/*1G genotype, and only 2 cases with *1G/*1G. For CYP3A5, there were 8 patients with *1/*1 genotype, 36 patients with *1/*3 genotype, and 56 cases with *3/*3. For ABCB1 C1236T, there were 10 patients with 1236CC genotype, 44 patients with 1236CT genotype, and 46 cases with 1236TT. For ABCB1 G2677 T/A, there were 13 patients with 2677 GG genotype, 57 patients with 2677 GT/GA genotype, and 30 cases with 2677 TT/AA/TA. In 100 patients, the frequencies of CYP3 A4*1G, CYP3 A5*3, ABCB1 C1236T and ABCB1 G2677 T/A mutant allele were 23%, 74%, 68% and 58.5%, respectively (Table 4). Through Hardy-Weinberg inspection and analysis, the results showed that the frequency of each gene reached genetic equilibrium (*P* > 0.05). The study data could be representative of the whole group.

**Table 4:** CYP3 A4, CYP3 A5 and ABCB1 genetic equilibrium test of genetic polymorphisms

SNP	Number of examples (theoretical)	Genotype frequency	Allele frequency	χ^2^	*P*
CYP3 A4					
*1/*1	56 (59.29)	0.56	*1 = 0.77	3.45	0.06
*1/*1G	42 (35.42)	0.42			
*1G/*1G	2 (5.29)	0.02	*1G = 0.23		
CYP3 A5					
*1/*1	8 (6.76)	0.08	*1 = 0.26	0.42	0.52
*1/*3	36 (38.48)	0.36			
*3/*3	56 (54.76)	0.56	*3 = 0.74		
ABCB1 C1236T					
CC	10 (10.24)	0.10	C = 0.32	0.012	0.91
CT	44(43.52)	0.44			
TT	46 (46.24)	0.46	*T* = 0.68		
ABCB1 G2677 T/A					
GG	13 (17.22)	0.13	G = 0.415	3.03	0.08
GT/A	57 (48.56)	0.57			
AA/TT/TA	30 (34.22)	0.30	T/A = 0.585		

### The effect of CYP3 A4, CYP3 A5, ABCB1, CCB and BMI on the clinical efficacy of TAC

Multivariate logistic regression analysis (Table 5) showed that the factors that influence the effect of clinical treatment included ABCB1 1236 mutant type TT (*P* = 0.018, OR = 12. 085, 95%CI 1.535–95.148), ABCB1 G2677 T/A gene mutation type TT or TA or AA (*P* = 0.042, OR = 8.683, 95%CI 1.080–69.819) and BMI (*P* = 0.020, OR = 15.838 95%CI 1.550–161.788) at the 0.05 level. For ABCB1 C1236T, TT genotype can increase the effectiveness of clinical treatment 12.085 times compared with CC and CT genotype. As for ABCB1 G2677 T/A, the clinical efficacy of patients with mutant genotype is 8.683 times than that of wild-type and heterozygous patient. Overweight patients can increase the clinical efficacy by 15.838 times than that of whose BMI was in normal range. However, CYP3A4, CYP3A5 and CCB do not directly affect the clinical efficacy of TAC (*P* = 0.125, *P* = 0.397, *P* = 0.357, respectively).

**Table 5:** The results of logistic regression analysis

Factors	β	SE	χ2	p	OR	95%CI
CYP3 A4	1.208	0.424	2.531	0.125	3.347	0.819–13.670
CYP3 A5	1.057	1.248	0.717	0.397	2.877	0.249–33.217
ABCB1 1236	2.492	1.053	5.603	0.018	12.085	1.535–95.148
ABCB1 2677	2.161	1.064	4.130	0.042	8.683	1.080–69.819
CCB	−1.349	1.464	0.849	0.357	0.260	0.015–4.572
BMI	2.762	1.186	5.428	0.020	15.838	1.550–161.788

### Adverse reactions

In the group of 100 patients receiving TAC treatment, adverse drug reactions occurred at 8 cases in different degrees, namely, 2 cases of patients with central nervous system response (hand tremor), 4 patients with elevated blood sugar and 2 patients with dyspnea. The incidence of side effects was about 8%.

## Discussion

We found evidence that ABCB1 C1236T, ABCB1 G2677 T/A and BMI are factors which may influence the clinical therapeutic effects of TAC on NS patients. We have not found that CYP3 A4, CYP3 A5 and whether the use of the CCB class antihypertensive drugs have impacts on the clinical treatment of patients with NS. These results indicate that detecting the above mentioned genes, especially ABCB1 C1236T and G2677 T/A, is important for the clinical implications of TAC, and that weight should also be taken into account. As a result, we should test genotypes in NS patients routinely prior to treatment to tailor treatment.

TAC, a kind of immunosuppressive drugs, can combine FK506 binding protein 12 (FKBP-12) and play a role in immune suppression [18, 19]. Pharmacokinetic characteristics of TAC were different among individuals, which could be influenced by the genetic factors such as single nucleotide polymorphisms (SNP), haplotype and DNA methylation [20]. In the CYP3A subfamily, CYP3 A4 and CYP3 A5 enzymes are responsible for TAC metabolism. P-gp encoded by the ABCB1 gene is responsible for transferring the drug from the cell to the outside [7, 12, 21]. Previous studies have focused on the effects of gene polymorphisms of CYP3 A4 and CYP3 A5 and ABCB1 genes on TAC metabolism in other setting and we have not found the relevant literature on NS. Therefore, more investigations should be performed to evaluate the effect of these related gene polymorphisms on the clinical efficacy of TAC in the treatment of NS patients.

ABCB1 gene is also known as multidrug resistance gene [8]. Some studies show that ABCB1 polymorphisms have nothing to do with the clinical efficacy of TAC [3, 9]. However, some studies have shown that ABCB1 1236 C > T can increase the blood concentration of TAC in the cell [7, 22]. Moreover, part of studies have come to the conclusion that the effect of TAC in patients with 2677G > T/A mutation genotype is better [4]. According to multi-factor logistic regression analysis in this study, we found that rs1128503 (1236C > T), rs2032582 (2677G > T/A) mutation carriers of these two loci were positively correlated with clinical efficacy of TAC in NS patients (Table 5).

We next try to interpret the mechanisms that ABCB1 C1236T and G2677 T/A genes affect the efficacy of TAC. ABCB1 1236C > T causes the codon to change from GGC to GGT. All of the genes encode glycine, which does not change the amino acid sequence of P-gp and belongs to synonymous mutation [21]. As we found that the mutated genotype of ABCB1 C1236T can increase the effectiveness compared with wild-type and heterozygous genotype, synonymous mutation might have a certain role in regulating the metabolism of TAC. ABCB1 2677G > T/A can result in 839 amino acid residue from alanine to threonine or serine, making it from lipotropy to hydrophily and then affecting the transport function of P-gp and protein expression [21]. As a result, the clinical efficacy of patients with mutant genotype of ABCB1 G2677 T/A was much more better than that of wild-type and heterozygous patients, which was observed in our present study.

A number of studies have shown that non-genetic factors such as body weight, drug interactions and the disease status of patients can also lead to individual differences in drug response [10, 12]. In this study, the dosage of TAC was calculated according to body weight. Patients with the same height and different weight were given different TAC dosage. The BMI of the patients in this study was in the normal range or above the normal range. We found that BMI is a factor affecting the clinical efficacy of patients with NS. Overweight patients can improve the clinical efficacy by 15.838 times than that of whose BMI is in normal range.

A meta-analysis suggested that the CYP3 A4*1G genetic polymorphism played an important role in renal transplant recipients and CYP3 A4*1/*1 carriers exhibited higher C_0_/Dose ratios than CYP3 A4*1G [14]. But some literatures indicated that the effect of CYP3 A4 genetic variability is not so well established [21]. Kurzawski et al. [10] finds that the CYP3A5 gene is an important factor in determining the dose requirement for TAC and individuals with CYP3A5*1 allele have higher TAC metabolism and lower blood TAC concentration. However, we found no significant correlation between CYP3 A4, CYP3 A5 genotype and clinical efficacy of TAC. The possible reasons are as follows. First, there may be other factors affecting the metabolism of CYP3A4 and CYP3A5 enzymes in patients with NS. Second, it is possible that the sample size is smaller, which leads to some deviation from the actual situation.

TAC plays a major role in clinical treatment by demethylation or hydroxylation of CYP450 enzyme. Thus, CYP450 enzyme inhibitors and inducers can also affect the TAC concentration and then have a certain impact on clinical efficacy. In our study, some patients took CCB antihypertensive drugs, which are a kind of CYP450 enzyme inhibitors, and did not take other CYP450 enzyme inhibitors and inducers, such as antibiotics, antifungal agents, antiviral drugs, psychiatric drugs and rifomycins [6]. In logistic regression, the application of CCB were used as covariates and clinical efficacy was used as dependent variable in this study. For the CCB, we concluded that the effect of it was not obvious in clinical treatment.

Previous studies showed that the mutation frequency of CYP3 A4 gene in Chinese population is about 30.8% [20]. In our present study, the mutation frequency was about 23%. The mutation frequency of CYP3 A5*3 is about 70% - 90% in Chinese population [11]. In the study, we found the CYP3A5 to be an unusual gene because the frequency of the variant allele (G) was higher (74%) than the wildtype (A) frequency (26%). 1236C > T ranges in allele frequency from 30 to 93% depending upon the ethnic population, and 2677G > T/A allele frequency varies as much as 2–65% among world population [22]. The frequencies of mutation in this study were 68% (1236C > T) and 58.5% (2677G > T/A), respectively.

We studied the effect of CYP3 A4, CYP3 A5, ABCB1 gene polymorphisms on the clinical efficacy of TAC in the treatment of NS and came up with the conclusion that ABCB1 C1236T and ABCB1 G2677 T/A could influence the clinical efficacy of TAC in the treatment of NS patients. However, there are also some limitations about the study. The inadequacy of this study is that the sample size is small, only 100 patients, and they are basically in the same region. There may be a linkage between the genes, which has a complicated effect on the pharmacokinetics of TAC, and the effects of various factors should be considered comprehensively. In the future, we need to expand the size of samples, confirm further study, and extend the time of follow-up.

## Conclusions

In summary, the results of this study reveal that the gene mutations of CYP3A4 and CYP3A5 and CCB may not directly affect the clinical efficacy of TAC. However, ABCB1 C1236T, ABCB1 G2677 T/A genotype and BMI are probably the factors influencing the clinical efficacy of TAC in treating patients with NS. Therefore, our study provides evidence that there may be a potential role for gene detection in tailoring therapy for patients with NS in order to improve response to treatment.

## Additional files

Additional file 1: CYP3 A4*1G gene sequencing map (forward sequencing). A: CYP3 A4 *1/*1 (wild type); B: CYP3 A4 *1/*1G (heterozygous type); C: CYP3 A4 *1G/*1G (mutant type). (TIFF 916 kb)

Additional file 2: CYP3 A5*1 gene sequencing map (forward sequencing). A: CYP3 A5 *1/*1 (wild type); B: CYP3 A5 *1/*3 (heterozygous type); C: CYP3 A5 *3/*3 (mutant type). (TIFF 1142 kb)

Additional file 3: ABCB1 C1236T gene sequencing map (reverse sequencing). A: ABCB1 1236 GG (wild type); B: ABCB1 1236 AG (heterozygous type); C: ABCB1 1236 AA (mutant type). (TIFF 833 kb)

Additional file 4: ABCB1 G2677T/A gene sequencing map (reverse sequencing). A: ABCB1 2677CC (wild type); B: ABCB1 2677CT (heterozygous type); C: ABCB1 2677CA (heterozygous type); D: ABCB1 2677TT (mutant type); E: ABCB1 2677AT (mutant type); F: ABCB1 2677AA (mutant type). (TIFF 1876 kb)

## Abbreviations

GFR: Glomerular filtration rate
NS: Nephrotic syndrome
P-gp: P-glycoprotein
SNP: Single Nucleotide Polymorphisms
TAC: Tacrolimus
