# Supplementary figures and images for: Effect of CYP3 A4, CYP3 A5 and ABCB1 gene polymorphisms on the clinical efficacy of tacrolimus in the treatment of nephrotic syndrome
Source: BMC Pharmacol Toxicol. 2018 Apr 3;19:14. doi: 10.1186/s40360-018-0202-9 (PMC5883590; doi:10.1186/s40360-018-0202-9)

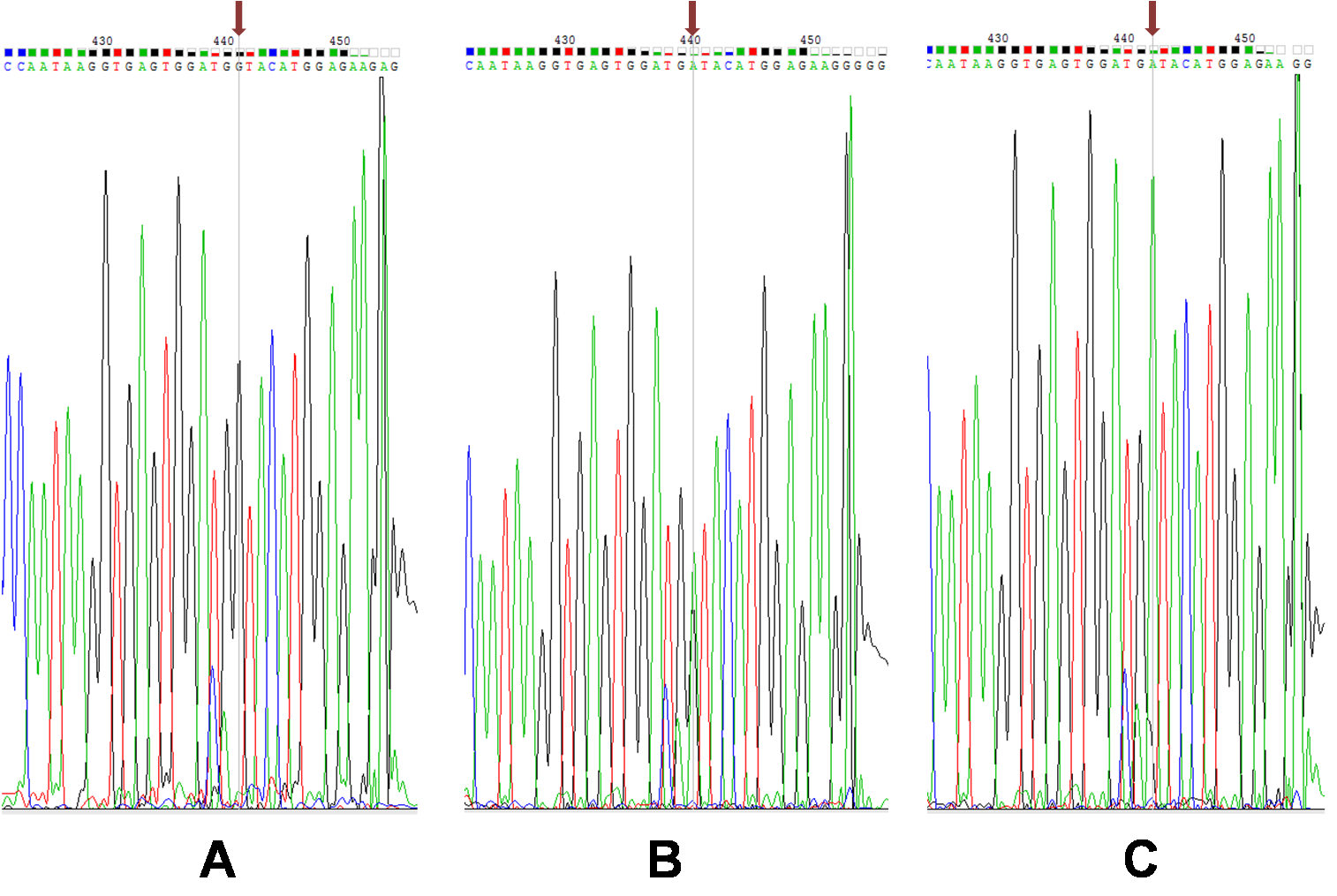

Supplement: Supplementary file 1 — CYP3 A4*1G gene sequencing map (forward sequencing). A: CYP3 A4 *1/*1 (wild type); B: CYP3 A4 *1/*1G (heterozygous type); C: CYP3 A4 *1G/*1G (mutant type). (TIFF 916 kb) [file 40360_2018_202_MOESM1_ESM.tif]

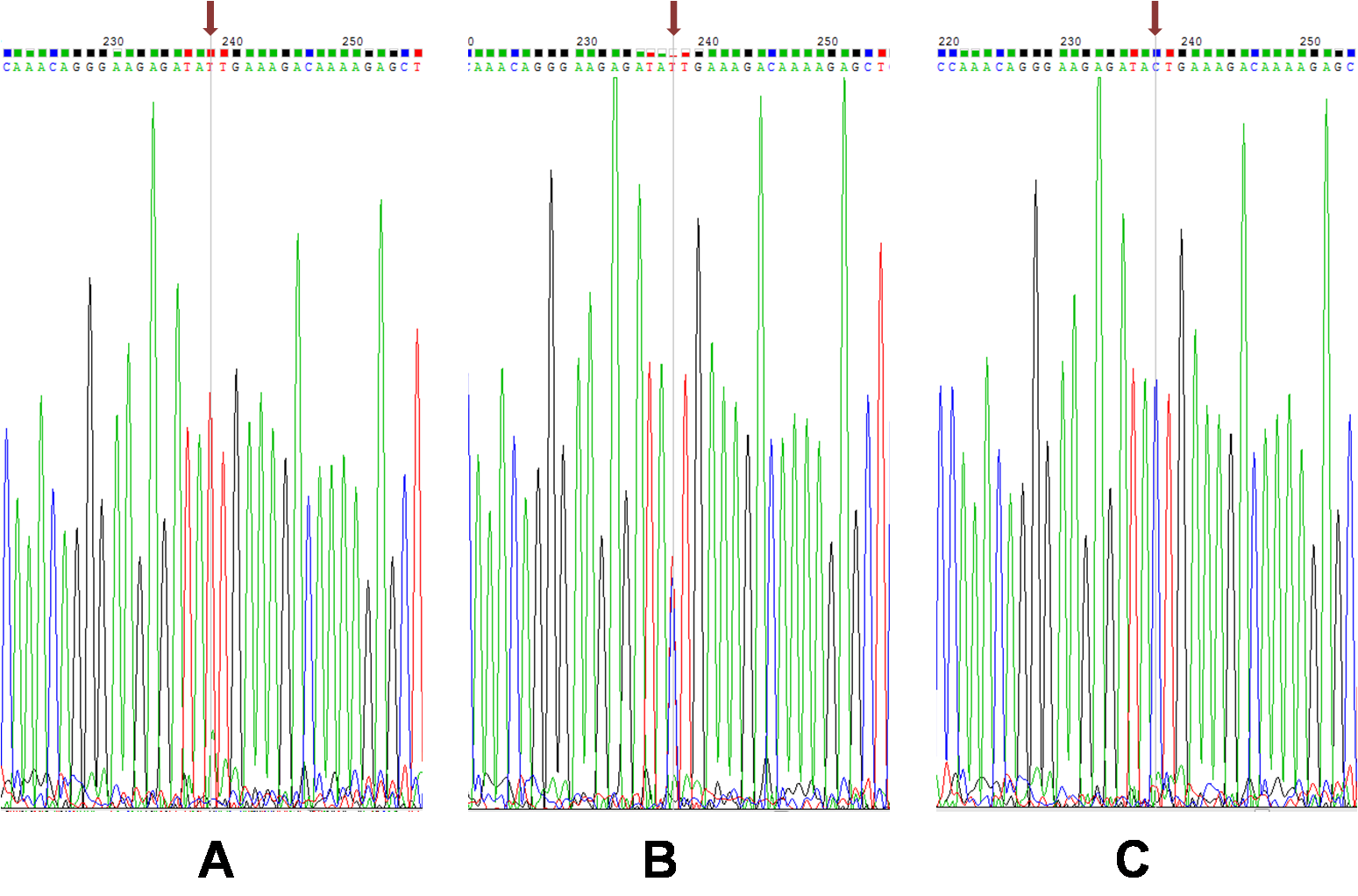

Supplement: Supplementary file 2 — CYP3 A5*1 gene sequencing map (forward sequencing). A: CYP3 A5 *1/*1 (wild type); B: CYP3 A5 *1/*3 (heterozygous type); C: CYP3 A5 *3/*3 (mutant type). (TIFF 1142 kb) [file 40360_2018_202_MOESM2_ESM.tif]

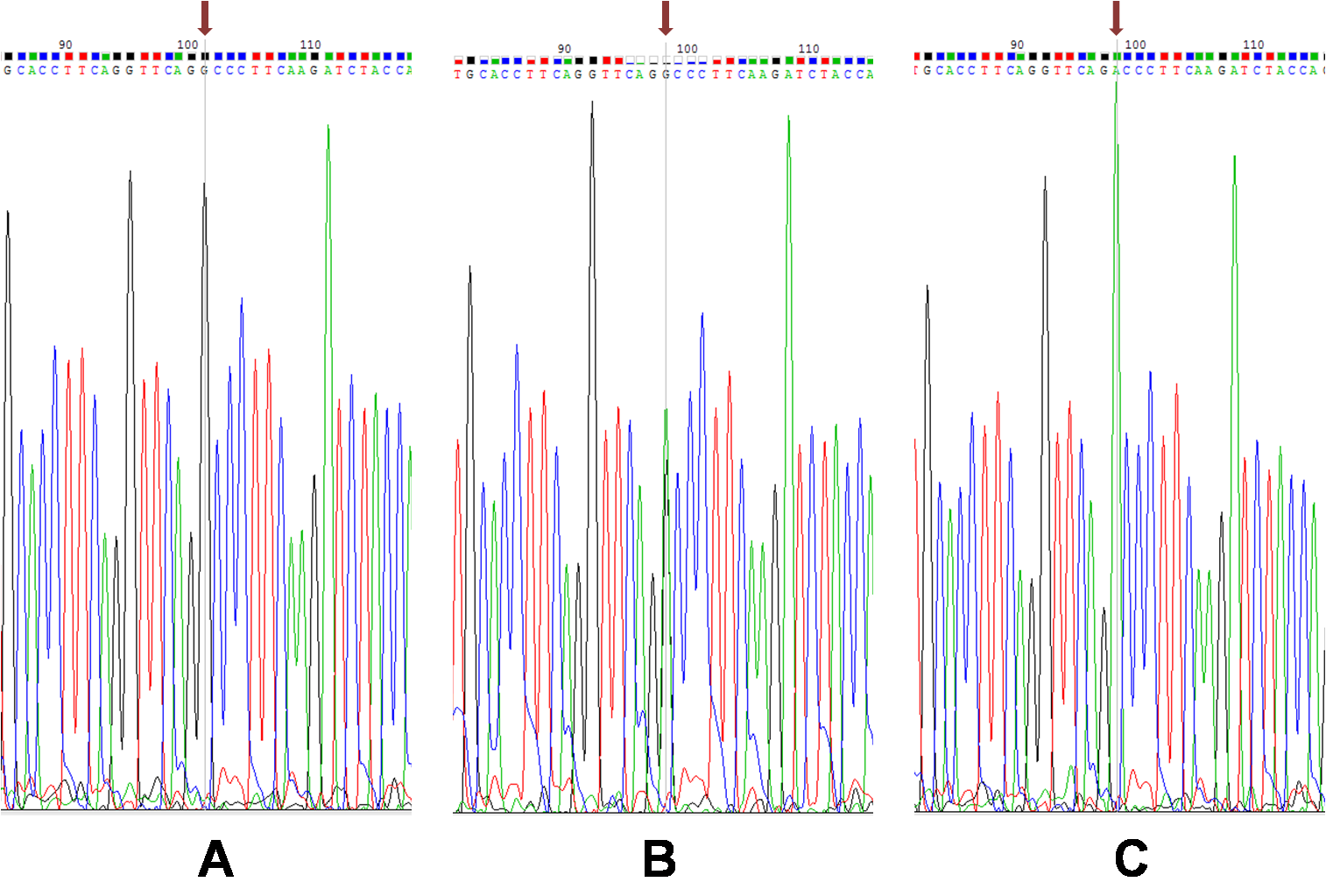

Supplement: Supplementary file 3 — ABCB1 C1236T gene sequencing map (reverse sequencing). A: ABCB1 1236 GG (wild type); B: ABCB1 1236 AG (heterozygous type); C: ABCB1 1236 AA (mutant type). (TIFF 833 kb) [file 40360_2018_202_MOESM3_ESM.tif]

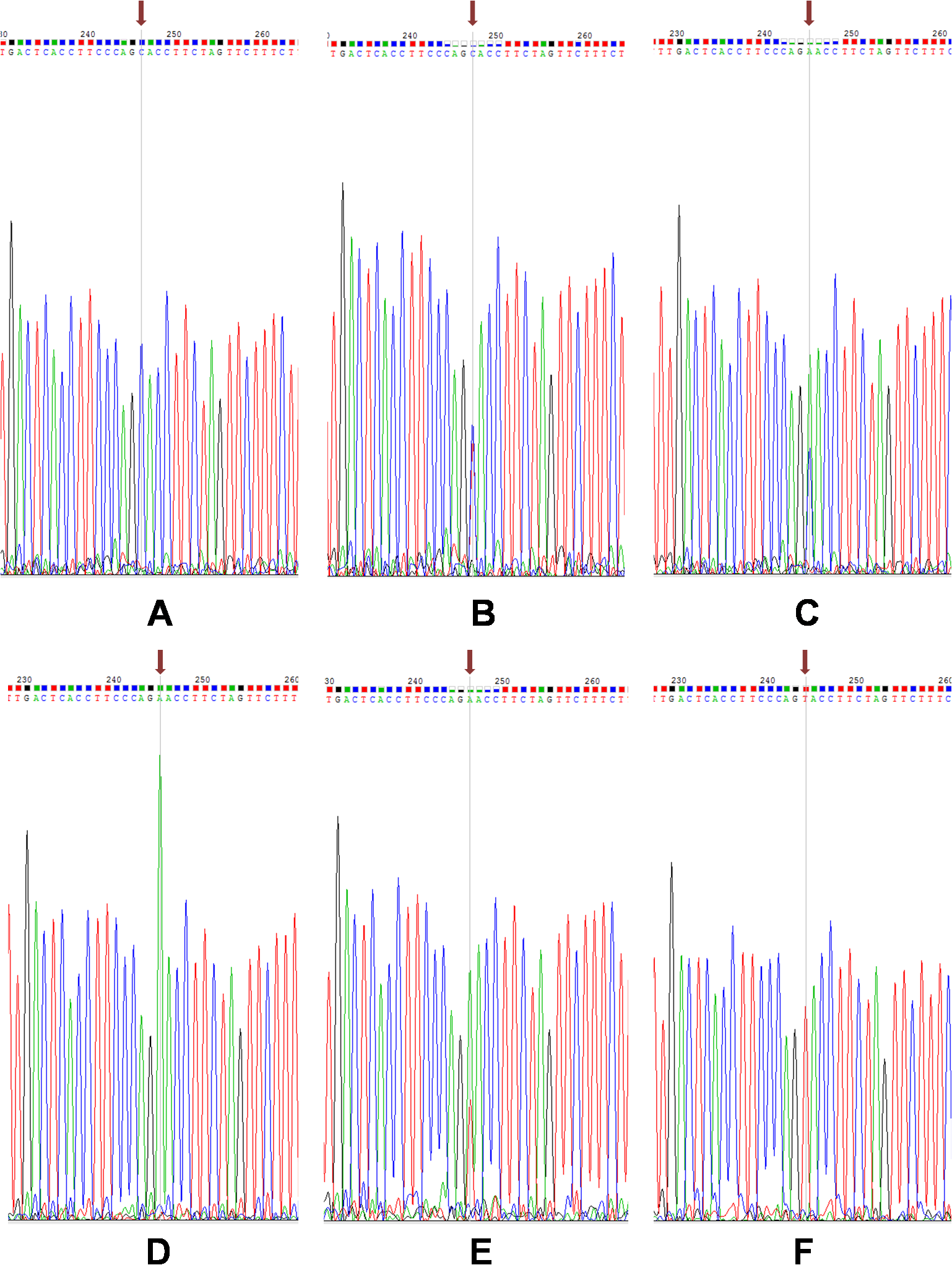

Supplement: Supplementary file 4 — ABCB1 G2677T/A gene sequencing map (reverse sequencing). A: ABCB1 2677CC (wild type); B: ABCB1 2677CT (heterozygous type); C: ABCB1 2677CA (heterozygous type); D: ABCB1 2677TT (mutant type); E: ABCB1 2677AT (mutant type); F: ABCB1 2677AA (mutant type). (TIFF 1876 kb) [file 40360_2018_202_MOESM4_ESM.tif]
